# Prolonged Monkeypox Virus Infections, California, USA, May 2022–August 2024

**DOI:** 10.3201/eid3110.250507

**Published:** 2025-10

**Authors:** Samuel Schildhauer, Kayla Saadeh, Robert E. Snyder, Eric C. Tang, Eric Chapman, Deanna A. Sykes, Philip Peters, Kathleen Jacobson, Jessica Watson, Kelly A. Johnson

**Affiliations:** University of California School of Medicine, Davis, California (S. Schildhauer); California Department of Public Health, Richmond, California, USA (S. Schildhauer, K. Saadeh, E.C. Tang, K. Jacobson, J. Watson, K.A. Johnson); California Department of Public Health, Sacramento, California, USA (R.E. Snyder, E. Chapman, D.A. Sykes, P. Peters); University of California, San Francisco, California, USA (K.A. Johnson)

**Keywords:** monkeypox, mpox, MPXV, viruses, zoonoses, California, United States, sexually transmitted infections, prolonged infection, epidemiology, HIV

## Abstract

Monkeypox virus (MPXV) infection typically lasts 14–28 days. Prolonged MPXV infection, in which symptoms or test positivity last >28 days, has been documented but not fully characterized. We used the California Department of Public Health (California, USA) mpox registry to compare prolonged (>28 days) and nonprolonged (<28 days) mpox cases by demographics, HIV status, and JYNNEOS vaccination status. Of 6,469 cases, 82 (1.3%) were prolonged. Persons with prolonged MPXV infections were more likely to be Black or African American (prolonged, 20.7%, vs. nonprolonged, 11.6%) and to have HIV (prolonged, 61.0%, vs. nonprolonged, 39.9%). Among persons with HIV, prolonged infections were more likely among those with lower (<200) CD4 counts (prolonged, 10.0%, vs. nonprolonged, 3.9%) or not engaged in HIV care (prolonged, 46.0%, vs. nonprolonged, 18.1%). No prolonged infections occurred in persons who received 2 JYNNEOS vaccine doses. Groups disproportionately affected by prolonged mpox should be prioritized for mpox vaccine education and outreach.

Mpox (formerly known as monkeypox) is a disease caused by the monkeypox virus (MPXV), a large, enveloped, double-stranded DNA orthopoxvirus that is closely related to smallpox (*1*). Although mpox has been recognized as endemic in West and Central Africa since 1970, occasional outbreaks have occurred outside of sub-Saharan Africa (*1*). In May 2022, an unprecedented global outbreak of MPXV (clade IIb) infections began, causing >102,000 cases by November 2024 (*2*,*3*); of those cases, 34,349 were reported in the United States and 6,643 were reported in the state of California (*2*,*4*). Because of the novel geographic spread of mpox, decreasing smallpox vaccine–induced immunity, and continuing endemic transmission in many countries around the world, a need exists to understand more about this virus, including viral transmission dynamics and clinical characteristics of infections (*5*,*6*).

MPXV infection typically causes self-limited systemic symptoms (e.g., fever, fatigue, and lymphadenopathy) and a rash (*7*,*8*). Although the duration of MPXV viral DNA detection by lesion-based testing varies, the median is 14–28 days from initial lesion manifestation (*8*–*11*). Although uncommon, some persons may experience prolonged MPXV infections, in which viral DNA is detected for >28 days, lesions persist for >28 days, or both (*12*–*15*), and previous publications have suggested that lesions can persist for up to 205 days (*15*,*16*). Persons with immunocompromising conditions, especially HIV infection, are at increased risk for severe mpox manifestations, including hospitalization (*11*,*17*). Case studies of clinically severe MPXV infections in immunocompromised persons demonstrate that more severe infections often occur in tandem with prolonged infections (*11*,*17*). However, clinically mild MPXV infections also can be prolonged in immunocompromised persons, suggesting the ability of their immune systems (perhaps combined with medical interventions) to partially control but not clear the virus (*11*,*12*,*14*,*17*).

Persons experiencing prolonged infections represent an epidemiologically and clinically important group; not only do they suffer from prolonged pain and a heightened risk for severe symptoms or hospitalization compared with persons in whom mpox symptoms do not last as long, but they also are infectious for longer periods and may present opportunities for more frequent mutations of MPXV (*12*,*16*,*18*). Understanding the prevalence of prolonged MPXV infections and the sociodemographic and clinical characteristics associated with persons who have prolonged infections can inform clinical care and public health policy, which could include expanding vaccination and messaging efforts. For these reasons, we sought to identify and characterize prolonged mpox cases in California by using the California Department of Public Health (CDPH) mpox case registry. We compared demographic and clinical characteristics between persons with prolonged and nonprolonged MPXV infections to identify populations who may be at a higher risk for prolonged MPXV infection.

## Methods

### Data Sources

All reactive orthopoxvirus or MPXV results are reportable to CDPH per statute. We matched California’s mpox surveillance registry to the statewide HIV surveillance registry to obtain HIV status and related clinical information. We also matched the mpox surveillance registry to the California Immunization Registry to determine status of vaccination with the 2-dose JYNNEOS (https://jynneos.com) mpox vaccine.

### Definitions

Given the abundance of literature stating that the typical clinical course of mpox lasts 14–28 days (*7*–*11*), we defined prolonged mpox cases as MPXV infections lasting >28 days between either 2 positive MPXV diagnostic PCR results or self-reported onset of symptoms and a subsequent positive MPXV diagnostic PCR result. Information on symptoms associated with disease onset and progression, including rash, fever, chills, lymphadenopathy, pruritis, and conjunctivitis, were reported by patients and documented by their healthcare providers. We defined nonprolonged mpox cases as illness in persons infected with MPXV who did not have a 28-day interval between either 2 positive test results or symptom onset and a positive test result. CDPH assesses possible MPXV reinfections through clinical follow-up and epidemiologic investigation; we excluded from this analysis all persons with probable MPXV reinfection, as defined by the Centers for Disease Control and Prevention (CDC) and confirmed by CDPH (*7*). We calculated the duration of infection for each mpox case as the amount of time in days between reported onset of symptoms or initial MPXV-positive PCR result and the last recorded MPXV-positive PCR result.

All mpox patients self-reported data on sex, race or ethnicity, and sexual orientation. Among patients with HIV, we aggregated CD4 lymphocyte counts into ranges (<200, 200–499, or >500 cells/mm^3^), consistent with the CDC classification system for HIV infection (*19*,*20*). We also aggregated HIV viral load as <200 or >200 copies/mL of blood. We limited CD4 and HIV viral load analyses to persons with HIV (PWH) and MPXV co-infection for whom these datapoints were available within 365 days before the earliest of either mpox symptom onset or testing date. PWH with no CD4 or viral load laboratory results within the last year may be out of HIV care and, as a result, more likely to be immunocompromised because of HIV infection; therefore, we considered them in a separate category. We classified JYNNEOS vaccination status as unvaccinated, 1-dose vaccinated, 2-dose vaccinated, or postexposure vaccinated (if the vaccine was administered in the 13 days before symptom onset date or first specimen collection date of their MPXV infection, in which case, based on the incubation period for mpox, we assumed they had a recent exposure and had received the JYNNEOS vaccine as postexposure prophylaxis). 

We modified an established mpox symptom severity score by Zucker et al. (*21*) to include data available in the CDPH mpox case registry (e.g., the degree of rash spread around the body, the number of mucosal sites affected, and the level of care required for the case) (Appendix Table 1). We did not include 3 of the severity score parameters from Zucker et al. (*21*) because of a lack of data availability (i.e., confluent lesions with diameter >2 cm, treatment for bacterial superinfection, and pain or analgesia requirement). The severity score uses a point system, whereby points accumulate for the severity of each of these categories; our modified severity score can result in a minimum of 0 points and a maximum of 15.

### Analysis

We analyzed prolonged and nonprolonged mpox cases in California that occurred during May 12, 2022–August 31, 2024, by comparing the case-patients’ demographic and clinical characteristics, JYNNEOS vaccination status, and HIV status (including CD4 counts and viral loads). We calculated percentages for each category among prolonged, nonprolonged, and total cases. To investigate the possible effect of HIV status on the association between race or ethnicity and prolonged mpox cases, we stratified race or ethnicity by HIV status (Appendix Table 2). We then calculated 95% CIs by using the exact Clopper–Pearson method for prolonged mpox cases (because of small cell sizes), and we used the Wilson method elsewhere. We calculated 95% CIs for age and severity score by using the Wald method. We considered nonoverlapping 95% CIs between prolonged and nonprolonged mpox cases as an indication of statistical significance for a given category.

Records with a missing illness onset date often had more missing values across all variables. Accordingly, we conducted a sensitivity analysis to see if results changed after excluding cases with missing onset dates. We did not conduct sensitivity analyses on data pertaining to HIV. We performed all analyses by using R version 4.3.2 (The R Project for Statistical Computing, https://www.r-project.org).

### Ethics

We conducted our study as an activity of public health surveillance. Therefore, the California Health and Human Services Agency Committee for the Protection of Human Subjects determined that our analysis was not human subjects research.

## Results

Overall, 6,469 mpox cases were reported in California during May 12, 2022–August 31, 2024. Most mpox patients self-identified as male (6,093 [94.2%]) and gay, lesbian, or same-gender loving (3,998 [61.8%]); 41.9% (2,710) self-identified as Hispanic or Latino. A total of 82 (1.3%) prolonged cases and 6,387 (98.7%) nonprolonged cases were reported. The longest duration of infection identified was 345 days; 21 (25.6%) of the 82 prolonged infections lasted >50 days.

In terms of sociodemographic characteristics, a larger percentage of patients with prolonged infections (20.7% [95% CI 12.57%–31.11%]; 17/82) identified as Black or African American compared with patients with nonprolonged infections (11.6% [95% CI 10.84%–12.41%]; 741/6,387), a finding that persisted after we stratified for HIV status (Appendix Table 2). Although 95% CIs overlapped among persons experiencing homelessness, a notably higher percentage of patients with prolonged infections also reported having experienced homelessness while infected with MPXV (9.8% [95% CI 4.31%–18.32%]; 8/82) compared with patients with nonprolonged infections (4.2% [95% CI 3.7–4.68]; 266/6,387). We observed no statistically significant differences in age, sex, or sexual orientation between patients with prolonged versus nonprolonged mpox infections (Table 1).

**Table 1:** Demographic characteristics of 6,469 patients with prolonged and nonprolonged monkeypox virus infections, California, USA, May 12, 2022–August 31, 2024*

Characteristic	Prolonged			Nonprolonged			Total	
	No.	% (95% CI)		No.	% (95% CI)		No.	% (95% CI)
Sex								
M	76	92.7 (84.75–97.27)		6,017	94.2 (93.61–94.75)		6,093	94.2 (93.59–94.73)
F	3	3.7 (0.76–10.32)		153	2.4 (2.05–2.8)		156	2.4 (2.06–2.81)
No option specified	2	2.4 (0.3–8.53)		150	2.3 (2–2.75)		152	2.3 (2.01–2.75)
Declined to answer or unknown	1	1.2 (0.03–6.61)		67	1.0 (0.83–1.33)		68	1.1 (0.83–1.33)
Sexual orientation								
Gay or lesbian	49	59.8 (48.34–70.44)		3,949	61.8 (60.63–63.01)		3,998	61.8 (60.61–62.98)
Bisexual	10	12.2 (6.01–21.29)		558	8.7 (8.07–9.45)		568	8.8 (8.11–9.49)
Heterosexual	8	9.8 (4.31–18.32)		504	7.9 (7.25–8.58)		512	7.9 (7.28–8.6)
Declined to answer	4	4.9 (1.34–12.02)		190	3.0 (2.59–3.42)		194	3.0 (2.61–3.44)
Orientation not listed	0	0.0 (0.0–4.4)		112	1.8 (1.46–2.11)		112	1.7 (1.44–2.08)
Unknown	11	13.4 (6.89–22.74)		1,074	16.8 (15.92–17.75)		1,085	16.8 (15.88–17.7)
Race or ethnicity								
Hispanic or Latino	36	43.9 (32.96–55.3)		2,674	41.9 (40.66–43.08)		2,710	41.9 (40.69–43.1)
White	19	23.2 (14.56–33.8)		1,867	29.2 (28.13–30.36)		1,886	29.2 (28.06–30.27)
Black or African American†	17	20.7 (12.57–31.11)		741	11.6 (10.84–12.41)		758	11.7 (10.96–12.52)
Asian	1	1.2 (0.03–6.61)		349	5.5 (4.93–6.05)		350	5.4 (4.89–5.99)
Other	2	2.4 (0.3–8.53)		122	1.9 (1.6–2.28)		124	1.9 (1.61–2.28)
Multiple races	0	0.0 (0.0–4.4)		87	1.4 (1.11–1.68)		87	1.3 (1.09–1.66)
AI/AN	1	1.2 (0.03–6.61)		23	0.4 (0.24–0.54)		24	0.4 (0.25–0.55)
NHOPI	0	0.0 (0.0–4.4)		29	0.5 (0.32–0.65)		29	0.4 (0.31–0.64)
Unknown	6	7.3 (2.73–15.25)		495	7.8 (7.12–8.43)		501	7.7 (7.12–8.42)
Housing status								
Homeless	8	9.8 (4.31–18.32)		266	4.2 (3.7–4.68)		274	4.2 (3.77–4.75)
Not homeless	36	43.9 (32.96–55.3)		2,286	35.8 (34.62–36.98)		2,322	35.9 (34.73–37.07)
Declined to answer	2	2.4 (0.3–8.53)		90	1.4 (1.15–1.73)		92	1.4 (1.16–1.74)
Unknown	36	43.9 (32.96–55.3)		3,745	58.6 (57.42–59.84)		3781	58.4 (57.24–59.64)
Mean age, y (95% CI)	38.32 (37.24–39.39)			37.16 (37.04–37.29)			3.29 (3.27–3.31)	
Total	82			6,387			6,469	

*AI/AN, American Indian or Alaska Native; IQR, interquartile range; NHOPI, Native Hawaiian or other Pacific Islander. †Indicates nonoverlapping 95% CIs (statistical significance).

Regarding clinical characteristics, among all 6,649 patients with mpox in California during the study period, 306 (4.7%) were hospitalized, 5,534 (85.5%) had not been vaccinated with the JYNNEOS vaccine, 80 (1.2%) were fully vaccinated with 2 JYNNEOS doses, and 2,599 (40.2%) were PWH (Table 2). The mean severity score among all case-patients was 3.29.

**Table 2:** Clinical characteristics of 6,469 patients with prolonged and nonprolonged monkeypox virus infections, California, USA, May 12, 2022–August 31, 2024

Characteristic	Prolonged			Nonprolonged			Total	
	No.	% (95% CI)		No.	% (95% CI)		No.	% (95% CI)
Hospitalization								
Yes*	21	25.6 (16.6–36.44)		285	4.5 (3.98–5.0)		306	4.7 (4.24–5.27)
No*	49	59.8 (48.34–70.44)		4,889	76.5 (75.49–77.57)		4,938	76.3 (75.28–77.35)
Unknown	12	14.6 (7.8–24.17)		1,213	19.0 (18.05–19.97)		1,225	18.9 (18–19.91)
JYNNEOS vaccination status								
Unvaccinated	76	92.7 (84.75–97.27)		5,458	85.5 (84.57–86.3)		5,534	85.5 (84.67–86.38)
1-dose vaccinated	2	2.4 (0.3–8.53)		287	4.5 (4.01–5.03)		289	4.5 (3.99–5.0)
Postexposure vaccinated	4	4.9 (1.34–12.02)		562	8.8 (8.13–9.52)		566	8.7 (8.09–9.46)
2-dose vaccinated	0	0.0 (0.0–4.4)		80	1.3 (1.01–1.56)		80	1.2 (0.99–1.54)
HIV status								
HIV-positive*	50	61.0 (49.57–71.56)		2,549	39.9 (38.71–41.12)		2,599	40.2 (38.99–41.38)
HIV-negative*	32	39.0 (28.44–50.43)		3,838	60.1 (58.88–61.29)		3,870	59.8 (58.62–61.01)
Mean symptom severity score (95% CI)	3.51 (3.3–3.72)			3.28 (3.26–3.31)			3.29 (3.27–3.31)	
Total	82			6,387			6,469	

*Indicates nonoverlapping 95% CIs (statistical significance).

A larger percentage of patients with prolonged mpox infections were hospitalized (25.6% [95% CI 16.6%–36.44%]; 21/82) compared with patients with nonprolonged infections (4.5% [95% CI 3.98%–5.0%]; 285/6,387). We observed no statistically significant differences between the severity score or vaccination status of patients with prolonged versus nonprolonged infections, but no patients with prolonged infection had received 2 doses of the JYNNEOS vaccine. A higher percentage of prolonged MPXV infections occurred in PWH (61.0% [95% CI 49.57%–71.56%]; 50/82) compared with nonprolonged MPXV infections (39.9% [95% CI 38.71%–41.12%]; 2,549/6,387). Among all mpox infections in PWH, most were in patients with a CD4 count >500 cells/mm^3^ (1,488/2,599 [57.3%]) and a viral load <200 copies/mL (1,857/2,599 [71.5%]) (Table 3).

**Table 3:** CD4 count and viral load among 2,599 persons with HIV among patients with prolonged and nonprolonged monkeypox virus infections, California, USA, May 12, 2022–August 31, 2024

Value	Prolonged			Nonprolonged			Total	
	No.	% (95% CI)		No.	% (95% CI)		No.	% (95% CI)
CD4 count								
<200	5	10.0 (3.33–21.81)		100	3.9 (3.24–4.75)		105	4.0 (3.35–4.87)
200–499	8	16.0 (7.17–29.11)		514	20.2 (18.65–21.77)		522	20.1 (18.59–21.67)
>500*	14	28.0 (16.23–42.49)		1,474	57.8 (55.9–59.73)		1,488	57.3 (55.34–59.14)
Out of care*	23	46.0 (31.81–60.68)		461	18.1 (16.64–19.63)		484	18.6 (17.17–20.17)
Viral load								
<200*	21	42.0 (28.19–56.79)		1,836	72.0 (70.25–73.74)		1,857	71.5 (69.68–73.15)
>200	6	12.0 (4.53–24.31)		228	8.9 (7.9–10.12)		234	9.0 (7.96–10.17)
Out of care*	23	46.0 (31.81–60.68)		485	19.0 (17.55–20.6)		508	19.5 (18.07–21.12)
Total	50			2,549			2,599	

*Indicates nonoverlapping 95% CIs (statistical significance).

Among PWH, a significantly higher percentage of patients with prolonged mpox infections had a CD4 count <200 (10.0% [95% CI 3.33%–21.81%]; 5/50) or had no CD4 laboratory results available (46.0% [95% CI 31.81%–60.68%]; 23/50) compared with patients with nonprolonged infections, among whom only 3.9% (95% CI 3.24%–4.75%; 100/2,549) had a CD4 count <200 and 18.1% (95% CI 16.64%–19.63%; 461/2,549) had no CD4 laboratory results available. In terms of viral suppression, a significantly lower percentage of patients with prolonged infections (42.0% [95% CI 28.19%–56.75%]; 21/50) had an HIV viral load <200 compared with patients with nonprolonged infections (72.0% [95% CI 70.25%–73.74%]; 1,836/2,549). Furthermore, a significantly higher percentage of patients with prolonged infections (46.0% [95% CI 31.81%–60.68%]; 23/50) had no HIV viral load information available compared with patients with nonprolonged infections (19.0% [95% CI 17.55%–20.6%]; 485/2,549).

We determined duration of MPXV infection by using the period between symptom onset date to specimen collection date for most mpox cases (5,360/6,469 [82.9%]). Information on illness onset date was missing for 1,109 (17.15%) mpox cases, so we calculated the duration of infection by using the period between positive MPXV PCR test results. Results from a sensitivity analysis indicated that excluding cases with a missing illness onset date did not affect the significance of any findings (Appendix Table 3, 4).

## Discussion

As MPXV continues to circulate worldwide, the epidemiologic and clinical features of prolonged MPXV infection remain poorly understood. In California, during May 2022–August 2024, prolonged MPXV infections were rare: only 82 persons (1.27%) were found to have MPXV infections for >28 days. Although more patients with prolonged infections were hospitalized than were patients with nonprolonged infections, most persons with prolonged infections reported mild symptoms, and patients with prolonged and nonprolonged cases had comparable symptom severity scores. Prolonged MPXV infections disproportionately affected Black or African American persons, persons experiencing homelessness, and PWH (especially PWH with uncontrolled HIV or who may be out of care). All 3 findings merit further consideration.

First, when analyzing demographic characteristics, we found that patients with prolonged MPXV infections disproportionately identified as Black or African American compared with patients with nonprolonged infections, an observation that remained consistent when we stratified by HIV status. Those results align with prior analyses in California and across the United States, which have shown that the incidence of mpox in general has been higher among Black or African American persons, who also were less likely to be vaccinated with the JYNNEOS vaccine than those from other racial or ethnic groups (*4*,*22*,*23*). An analysis of an mpox outbreak in Los Angeles, California, for example, found that no Black or African American persons with mpox were fully vaccinated with the JYNNEOS vaccine, compared with 57.0% of White persons with mpox (*23*). Those differences, at least in part, probably are influenced by social determinants of health, including inadequate access to health care, leading to poor vaccination uptake and a higher risk for not only acquiring mpox but also having more prolonged infections (*24*,*25*), as we observed in our analysis.

Second, a disproportionate number of prolonged cases occurred in PWH, especially among persons with low CD4 counts and persons who lacked HIV laboratory testing in the preceding 12 months, which may signal that a person is out of HIV care (*26*). For many persons who are on antiretroviral therapy and have well-controlled HIV, MPXV infections are mild and without serious sequelae (*19*). However, PWH who are not virologically suppressed may experience prolonged illness, debilitating illness, or both because of MPXV infection (*14*–*16*), a finding confirmed by our study. Our results indicate that clinicians should remain vigilant to the possibility of prolonged MPXV infection in persons with persistent symptoms, particularly persons with advanced HIV infection. Those persons also represent a group for whom MPXV antiviral and immunoglobulin therapies may be considered to prevent or treat severe or prolonged MPXV infection, although the effectiveness of those therapies is still being researched (*11*,*14*,*15*,*27*).

Third, a notably higher proportion of prolonged mpox infections occurred in persons who were experiencing homelessness, regardless of whether they were living with HIV, although overlapping 95% CIs indicate some uncertainty around those estimates. The relationship between social determinants of health (e.g., housing status) and MPXV infection may follow that of other infectious diseases, in which persons experiencing homelessness are at increased risk for contracting infectious diseases and having more severe disease (*28*,*29*). Such populations often consist of persons who have underlying health conditions and experience worse health outcomes because of limited access to healthcare (including vaccination) that is driven at least in part by housing insecurity (*28*–*31*). Dedicated outreach for such groups, including interventions such as mobile vans and street medicine teams with the ability to offer mpox education and vaccination, may be helpful.

Regarding mpox prevention, the 2-dose JYNNEOS vaccine series prevents severe mpox disease and reduces the odds of hospitalization among persons with mpox compared with persons who are unvaccinated (*32*). We observed no prolonged infections among persons who had received 2 doses of JYNNEOS vaccine in our sample, which is additional evidence that getting the 2 recommended vaccine doses protects against severe disease, including prolonged infections. Persons who are at risk for adverse outcomes related to MPXV infection, including prolonged infections, such as Black or African American persons, gay persons, bisexual persons, men who have sex with men, and PWH, especially those with advanced HIV infection, thus should be prioritized for community-based vaccine education and outreach (*27*,*33*,*34*).

One limitation of our analysis is that we might have underestimated the duration of prolonged MPXV infections because that variable was dependent on having either a reported symptom onset date or a person having been tested multiple times for MPXV, which is not routine in California. Similarly, we might have underestimated the number of prolonged infections if healthcare providers did not obtain repeat MPXV testing of patients with ongoing symptoms or rashes. Prolonged infections may be overrepresented among persons with inadequate access to healthcare, given that these groups may be less likely to seek care for mild illness because of structural barriers and less favorable social determinants of health, which have been linked to worse health outcomes in various other contexts (*24*,*25*,*29*,*35*). In addition, mpox cases in persons who were hospitalized or were experiencing homelessness, living in congregate settings (e.g., skilled nursing facilities or shelters), or both could have been overrepresented among persons with prolonged MPXV infections because they may be more likely to be tested multiple times. Our analysis also was subject to missing information; data that contributed to the severity score, such as lesion prevalence, were lacking for multiple cases, leading to the possible underestimation of severity and possibly decreasing the metric’s utility. As a result, the mean severity score of 3.51 among prolonged and 3.28 among nonprolonged mpox cases primarily came from 1 point in active lesion prevalence (1–9 lesions), 1 point in extent of body involvement (1–3 parts), and 1 point for use of outpatient services. Our analysis could not differentiate between hospitalizations that occurred in intensive care units and those that did not. The use of overlapping 95% CIs as an indicator for statistical significance instead of p values is conservative and might have led to underestimation of statistical differences in certain circumstances. We also could not assess orthopoxvirus antiviral use among prolonged cases compared with nonprolonged cases because the primary mechanism for accessing antiviral therapy during the study period was through a blinded placebo-controlled clinical trial, in which patients’ assigned treatment groups were not reported to public health surveillance. In addition, passive surveillance systems may inadequately capture data on important confounding variables such as socioeconomic status or healthcare access. Furthermore, we could not evaluate the infectivity of persons with positive MPXV PCR results. Because a lesion is necessary for testing in the United States, it is presumed that all persons with prolonged infections had lesions; however, we did not evaluate whether the lesions contained replication-competent virus.

In conclusion, our analysis compared prolonged versus nonprolonged MPXV infections, characterizing persons who may be at increased risk for prolonged infections, which can improve clinical care and public health practice. During the study period, prolonged mpox cases were rare in California and were more common among persons who identified as Black or African American, persons who experienced homelessness, and in PWH, especially PWH who had uncontrolled HIV infection. Those groups, who often face barriers to healthcare access, should be prioritized for JYNNEOS vaccination, alongside other groups at risk for severe disease. Furthermore, ensuring that PWH who have mpox are adequately linked to ongoing HIV treatment may be an opportunity to reduce poor health outcomes, including prolonged mpox disease.

Appendix Additional information about prolonged monkeypox virus infections, California, USA, May 2022–August 2024.
