# Supplementary material for: Prolonged Monkeypox Virus Infections, California, USA, May 2022–August 2024
Source: Emerg Infect Dis. 2025 Oct;31(10):1935–41. doi: 10.3201/eid3110.250507 (PMC12483021; doi:10.3201/eid3110.250507)
Supplement: Appendix — Additional information about prolonged monkeypox virus infections, California, USA, May 2022–August 2024. [file 25-0507-Techapp-s1.pdf]

Article DOI: https://doi.org/10.3201/eid3110.250507

EID cannot ensure accessibility for supplementary materials supplied by authors. Readers who have difficulty accessing supplementary content should contact the authors for assistance.

Prolonged Monkeypox Virus Infections, California, USA, May 2022–August 2024

Appendix

Appendix Table 1. Mpox severity score grading system

Parameter	Grade 0 (0 points)	Grade 1 (1 points)	Grade 2 (2 points)	Grade 3 (3 points)	Grade 4 (4 points)
Active lesion burden, number (total)	0	1–9	10–99	>100	
Lesion burden, extent of body involvement (head/neck, chest/abdomen, back groin/buttocks/anus, left arm, left hand, right arm, right hand, left leg, left foot, right leg, right foot)	0	1–3	4–6	7–9	10–12
Mucosal areas affected (4 anatomic locations: anorectal, oropharyngeal, genital (solely mucosal), ocular lesion	0		1 location	2 locations	≥3 locations
Level of care		Outpatient	Inpatient, non-ICU related to Mpox	Inpatient, ICU related to Mpox	Death

Appendix Table 2. Race/ethnicity of prolonged vs nonprolonged mpox cases by HIV status, California, 2022–2024, n = 6469

	Prolonged		Nonprolonged		Total	
Race/ethnicity	n	% (95% CI)	n	% (95% CI)	n	% (95% CI)
People with HIV						
American Indian or Alaska Native	1	2.0 (0.05–10.65)	14	0.5 (0.33–0.92)	15	0.6 (0.35–0.95)
Asian	0	0.0 (0.0–7.11)	99	3.9 (3.2–4.71)	99	3.8 (3.14–4.62)
Black or African American	11	22.0 (11.53–35.96)	340	13.3 (12.07–14.71)	351	13.5 (12.24–14.87)
Hispanic or Latino	23	46.0 (31.81–60.68)	1188	46.6 (44.68–48.55)	1211	46.6 (44.68–48.52)
Multiple Races	0	0.0 (0.0–7.11)	33	1.3 (0.92–1.81)	33	1.3 (0.91–1.78)
Native Hawaiian or Other Pacific Islander	0	0.0 (0.0–7.11)	8	0.3 (0.16–0.62)	8	0.3 (0.16–0.61)
Other	1	2.0 (0.05–10.65)	47	1.8 (1.39–2.44)	48	1.8 (1.4–2.44)
Unknown	5	10.0 (3.33–21.81)	151	5.9 (5.07–6.91)	156	6.0 (5.15–6.98)
White	9	18.0 (8.58–31.44)	669	26.2 (24.57–27.99)	678	26.1 (24.43–27.81)
Total	50		2549		2599	
People without HIV						
American Indian or Alaska Native	0	0.0 (0.0–10.89)	9	0.2 (0.12–0.45)	9	0.2 (0.12–0.44)
Asian	1	3.1 (0.08–16.22)	250	6.5 (5.78–7.34)	251	6.5 (5.75–7.31)
Black or African American	6	18.8 (7.21–36.44)	401	10.5 (9.52–11.46)	407	10.5 (9.59–11.53)
Hispanic or Latino	13	40.6 (23.7–59.36)	1486	38.7 (37.2–40.28)	1499	38.7 (37.22–40.29)
Multiple Races	0	0.0 (0.0–10.89)	54	1.4 (1.08–1.83)	54	1.4 (1.07–1.82)
Native Hawaiian or Other Pacific Islander	0	0.0 (0.0–10.89)	21	0.5 (0.36–0.84)	21	0.5 (0.36–0.83)
Other	1	3.1 (0.08–16.22)	75	2.0 (1.56–2.44)	76	2.0 (1.57–2.45)
Unknown	1	3.1 (0.08–16.22)	343	8.9 (8.08–9.88)	344	8.9 (8.03–9.83)
White	10	31.2 (16.12–50.01)	1198	31.2 (29.78–32.71)	1208	31.2 (29.78–32.7)
Total	32		3837		3869	

*Indicates non-overlapping confidence intervals.

Appendix Table 3. Demographic characteristics of prolonged vs nonprolonged mpox infections excluding records with a missing illness onset date, California, 2022–2024 (n = 5360)

	Prolonged		Nonprolonged		Total	
Characteristic	n	% (95% CI)	n	% (95% CI)	n	% (95% CI)
Gender						
Male	72	94.7 (87.07–98.55)	5007	94.8 (94.12–95.33)	5079	94.8 (94.13–95.32)
Female	2	2.6 (0.32–9.18)	116	2.2 (1.83–2.63)	118	2.2 (1.84–2.63)
No option specified	2	2.6 (0.32–9.18)	129	2.4 (2.06–2.89)	131	2.4 (2.06–2.89)
Declined/Unknown	0	0.0 (0.0–4.74)	32	0.6 (0.43–0.85)	32	0.6 (0.42–0.84)
Sexual orientation						
Gay, lesbian, or same-gender loving	48	63.2 (51.31–73.94)	3675	69.5 (68.29–70.78)	3723	69.5 (68.21–70.68)
Bisexual	10	13.2 (6.49–22.87)	519	9.8 (9.05–10.65)	529	9.9 (9.1–10.7)
Heterosexual or straight	7	9.2 (3.78–18.06)	437	8.3 (7.56–9.04)	444	8.3 (7.58–9.05)
Declined to answer	3	3.9 (0.82–11.11)	171	3.2 (2.79–3.75)	174	3.2 (2.8–3.76)
Orientation not listed	0	0.0 (0–4.74)	84	1.6 (1.29–1.96)	84	1.6 (1.27–1.94)
Unknown	8	10.5 (4.66–19.69)	398	7.5 (6.85–8.28)	406	7.6 (6.9–8.31)
Race/ethnicity						
Hispanic or Latino	34	44.7 (33.31–56.59)	2335	44.2 (42.86–45.53)	2369	44.2 (42.87–45.53)
White	18	23.7 (14.68–34.82)	1590	30.1 (28.87–31.34)	1608	30.0 (28.79–31.24)
Black or African American*	17	22.4 (13.6–33.38)	607	11.5 (10.66–12.38)	624	11.6 (10.81–12.53)
Asian	1	1.3 (0.03–7.11)	306	5.8 (5.19–6.45)	307	5.7 (5.14–6.38)
Other	2	2.6 (0.32–9.18)	90	1.7 (1.39–2.09)	92	1.7 (1.4–2.1)
Multiple Races	0	0.0 (0.0–4.74)	81	1.5 (1.24–1.9)	81	1.5 (1.22–1.87)
American Indian or Alaska Native	1	1.3 (0.03–7.11)	20	0.4 (0.25–0.58)	21	0.4 (0.26–0.6)
Native Hawaiian or Other Pacific Islander	0	0.0 (0.0–4.74)	26	0.5 (0.34–0.72)	26	0.5 (0.33–0.71)
Unknown	3	3.9 (0.82–11.11)	229	4.3 (3.82–4.92)	232	4.3 (3.82–4.91)
Mean age (95% CI)	38.05	36.9–39.21	36.93	36.79–37.07	3.68	3.66–3.7
Person experiencing homelessness						
Yes homeless	7	9.2 (3.78–18.06)	223	4.2 (3.71–4.8)	230	4.3 (3.78–4.87)
Not homeless	36	47.4 (35.79–59.16)	2265	42.9 (41.54–44.2)	2301	42.9 (41.61–44.26)
Declined	1	1.3 (0.03–7.11)	51	1.0 (0.73–1.27)	52	1.0 (0.74–1.27)
Unknown	32	42.1 (30.86–53.98)	2745	51.9 (50.6–53.29)	2777	51.8 (50.47–53.15)
Total	76		5284		5360	

*Indicates non-overlapping confidence intervals.

Appendix Table 4. Clinical characteristics of prolonged vs. nonprolonged mpox cases excluding records with a missing illness onset date, California, 2022–2024 (n = 5360)

	Prolonged		Nonprolonged		Total	
Characteristic	n	% (95% CI)	n	% (95% CI)	n	% (95% CI)
Hospitalization						
Yes*	20	26.3 (16.87–37.68)	252	4.8 (4.23–5.38)	272	5.1 (4.52–5.7)
No*	49	64.5 (52.66–75.12)	4615	87.3 (86.42–88.21)	4664	87.0 (86.09–87.89)
Unknown	7	9.2 (3.78–18.06)	417	7.9 (7.2–8.65)	424	7.9 (7.22–8.66)
Vaccination status						
Unvaccinated*	72	94.7 (87.07–98.55)	4492	85.0 (84.02–85.95)	4564	85.1 (84.17–86.08)
One dose vaccinated	2	2.6 (0.32–9.18)	237	4.5 (3.96–5.08)	239	4.5 (3.94–5.04)
Post-exposure vaccinated	2	2.6 (0.32–9.18)	480	9.1 (8.34–9.89)	482	9.0 (8.26–9.79)
Two dose vaccinated	0	0.0 (0.0–4.74)	75	1.4 (1.13–1.78)	75	1.4 (1.12–1.75)
Severity score						
Mean (95% CI)	3.67	3.47–3.88	3.68	3.66–3.7	3.68	3.66–3.7
Total	76		5284		5360	

*Indicates non-overlapping confidence intervals.
